# 1′,3′,4′,5′,7′,8′-Hexafluoro-1,1′′,2,2′′,3,3′′,4,4′′-octa­phenyl-2′,6′-dihydro­dispiro­[cyclo­penta-1,3-diene-5,2′-naphthalene-6′,5′′-cyclo­penta-1′′,3′′-diene] dichloro­methane monosolvate

**DOI:** 10.1107/S1600536811030285

**Published:** 2011-07-30

**Authors:** Shuhong Li

**Affiliations:** a Department of Chemistry, School of Science, Beijing Technology and Business University, Beijing 100048, People’s Republic of China

## Abstract

The mol­ecule of the title compound, C_66_H_40_F_6_·CH_2_Cl_2_, is centrosymmetric; the dihedral angle between the central fluorinated unit and the cyclo­penta­diene ring is 88.36 (7)°. The dihedral angles between the cyclo­penta­diene ring and the four surrounding phenyl rings are in the range 26.6 (1)–65.6 (1)°. Centrosymmetric cavities in the crystal structure are populated by disordered dichloro­methane solvent mol­ecules.

## Related literature

For the synthesis of partially fluorinated polycyclic aromatic compounds, see: Cho *et al.* (2005 ▶); Morrison *et al.* (2005 ▶); Swartz *et al.* (2005 ▶); Wang *et al.* (2006 ▶); Chen *et al.* (2006 ▶); Tannaci *et al.* (2007 ▶). For a one-pot synthetic protocol for partially fluorinated acenes, see: Li *et al.* (2008 ▶).
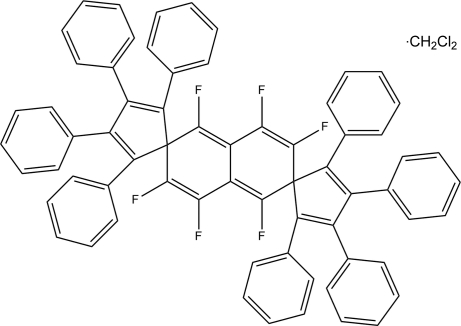


## Experimental

### 

#### Crystal data

C_66_H_40_F_6_·CH_2_Cl_2_
*M* *_r_* = 1031.91
Triclinic, 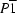

*a* = 9.1652 (18) Å
*b* = 11.814 (2) Å
*c* = 12.353 (3) Å
α = 79.67 (3)°
β = 73.38 (3)°
γ = 84.91 (3)°
*V* = 1259.9 (4) Å^3^
*Z* = 1
Mo *K*α radiation
μ = 0.20 mm^−1^
*T* = 173 K
0.28 × 0.26 × 0.18 mm

#### Data collection

Rigaku R-AXIS RAPID IP area-detector diffractometer
Absorption correction: multi-scan (*ABSCOR*; Higashi, 1995 ▶) *T*_min_ = 0.947, *T*_max_ = 0.966
10301 measured reflections
5748 independent reflections
4416 reflections with *I* > 2σ(*I*)
*R*_int_ = 0.073

#### Refinement

*R*[*F* ^2^ > 2σ(*F* ^2^)] = 0.092
*wR*(*F* ^2^) = 0.213
*S* = 1.15
5748 reflections
352 parameters
3 restraints
H-atom parameters constrained
Δρ_max_ = 0.36 e Å^−3^
Δρ_min_ = −0.42 e Å^−3^

### 

Data collection: *RAPID-AUTO* (Rigaku, 2001 ▶); cell refinement: *RAPID-AUTO*; data reduction: *RAPID-AUTO*; program(s) used to solve structure: *SHELXS97* (Sheldrick, 2008 ▶); program(s) used to refine structure: *SHELXL97* (Sheldrick, 2008 ▶); molecular graphics: *SHELXTL* (Sheldrick, 2008 ▶); software used to prepare material for publication: *SHELXL97*.

## Supplementary Material

Crystal structure: contains datablock(s) I, global. DOI: 10.1107/S1600536811030285/ld2019sup1.cif

Structure factors: contains datablock(s) I. DOI: 10.1107/S1600536811030285/ld2019Isup2.hkl

Additional supplementary materials:  crystallographic information; 3D view; checkCIF report

## Figures and Tables

**Fig. 1.**
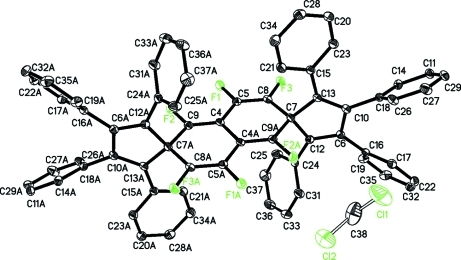
The molecular structure of (I) with solvate dichloromethane, showing atom labelling and 50% probability displacement ellipsoids for non-H atoms.

## supplementary crystallographic information

### Comment

Polycyclic aromatic acenes have received considerable attention in the past few decades due to their potential for construction of organic electronic devices. During the synthesis of the partially fluorinated polycyclic aromatic compounds, the title molecule was isolated as a byproduct. Its centrosymmetric molecular structure is shown in Fig. 1. The dihedral angle between the central naphthalene ring and cyclopenta diene ring is 88.36 (7)°. A molecule of solvate dichloromethane was found disordered over crystallographic inversion center.

### Experimental

A mixture of granular lithium (18.2 mg, 2.6 mmol) and naphthalene (336.3 mg, 2.6 mmol) in THF was stirred at room temperature for 4 h. To the resulting solution of lithium naphthalenide, a solution of diphenylacetylene (311.6 mg, 1.75 mmol) in THF (4 ml) was added at room temperature. After stirring for 20 min, perfluoronaphthalene (123 mg, 0.45 mmol) in THF (5 ml) was added to the reaction mixture at room temperature. The reaction mixture was stirred for 1 h and then quenched with a saturated aqueous solution of NH_4_Cl. The mixture was extracted with ethyl ether. The organic layer was washed with brine, dried over MgSO_4_, filtered, and concentrated under reduced pressure. The resulting mixture was gradually passed through a silica gel column with different ratio of petroleum ether/ethyl acetate mixture as an eluent, followed by further purification by recrystallization (CH_2_Cl_2_/acetone) yielding pale yellow crystals.

### Refinement

H atoms were located in a difference map and then were placed geometrically and refined using a riding model with *C*–*H*(dichloromethane) = 0.99Å and aromatic C–H = 0.95Å and with *U*_iso_(H) = 1.2 times *U*_eq_(C).

### Crystal data

**Table:**

C_66_H_40_F_6_·CH_2_Cl_2_	*Z* = 1
*M**_r_* = 1031.91	*F*(000) = 532
Triclinic, *P*1	*D*_x_ = 1.360 Mg m^−^^3^
*a* = 9.1652 (18) Å	Mo *K*α radiation, λ = 0.71069 Å
*b* = 11.814 (2) Å	Cell parameters from 17659 reflections
*c* = 12.353 (3) Å	θ = 1.8–27.5°
α = 79.67 (3)°	µ = 0.20 mm^−^^1^
β = 73.38 (3)°	*T* = 173 K
γ = 84.91 (3)°	Block, pale yellow
*V* = 1259.9 (4) Å^3^	0.28 × 0.26 × 0.18 mm

### Data collection

**Table:**

Rigaku R-AXIS RAPID IP area-detector diffractometer	5748 independent reflections
Radiation source: fine-focus sealed tube	4416 reflections with *I* > 2σ(*I*)
graphite	*R*_int_ = 0.073
ω scans at fixed χ = 45°	θ_max_ = 27.5°, θ_min_ = 2.3°
Absorption correction: multi-scan (*ABSCOR*; Higashi, 1995)	*h* = −11→11
*T*_min_ = 0.947, *T*_max_ = 0.966	*k* = −15→15
10301 measured reflections	*l* = −16→16

### Refinement

**Table:**

Refinement on *F*^2^	Primary atom site location: structure-invariant direct methods
Least-squares matrix: full	Secondary atom site location: difference Fourier map
*R*[*F*^2^ > 2σ(*F*^2^)] = 0.092	Hydrogen site location: inferred from neighbouring sites
*wR*(*F*^2^) = 0.213	H-atom parameters constrained
*S* = 1.15	*w* = 1/[σ^2^(*F*_o_^2^) + (0.0807*P*)^2^ + 0.7579*P*] where *P* = (*F*_o_^2^ + 2*F*_c_^2^)/3
5748 reflections	(Δ/σ)_max_ < 0.001
352 parameters	Δρ_max_ = 0.36 e Å^−^^3^
3 restraints	Δρ_min_ = −0.42 e Å^−^^3^

### Special details

**Table:**

Geometry. All e.s.d.'s (except the e.s.d. in the dihedral angle between two l.s. planes) are estimated using the full covariance matrix. The cell e.s.d.'s are taken into account individually in the estimation of e.s.d.'s in distances, angles and torsion angles; correlations between e.s.d.'s in cell parameters are only used when they are defined by crystal symmetry. An approximate (isotropic) treatment of cell e.s.d.'s is used for estimating e.s.d.'s involving l.s. planes.
Refinement. Refinement of *F*^2^ against ALL reflections. The weighted *R*-factor *wR* and goodness of fit *S* are based on *F*^2^, conventional *R*-factors *R* are based on *F*, with *F* set to zero for negative *F*^2^. The threshold expression of *F*^2^ > σ(*F*^2^) is used only for calculating *R*-factors(gt) *etc*. and is not relevant to the choice of reflections for refinement. *R*-factors based on *F*^2^ are statistically about twice as large as those based on *F*, and *R*- factors based on ALL data will be even larger.

### Fractional atomic coordinates and isotropic or equivalent isotropic displacement parameters (Å^2^)

**Table:**

	*x*	*y*	*z*	*U*_iso_*/*U*_eq_	Occ. (<1)
F1	0.1344 (2)	0.35667 (14)	0.68083 (15)	0.0317 (4)	
F2	0.0308 (2)	0.55799 (15)	0.71831 (14)	0.0308 (4)	
F3	0.18434 (19)	0.19572 (14)	0.54824 (15)	0.0301 (4)	
C4	0.0195 (3)	0.4898 (2)	0.5551 (2)	0.0206 (6)	
C5	0.0901 (3)	0.3773 (2)	0.5839 (2)	0.0224 (6)	
C6	0.2157 (3)	0.2222 (2)	0.2463 (2)	0.0215 (6)	
C7	0.0815 (3)	0.3093 (2)	0.4048 (2)	0.0221 (6)	
C8	0.1140 (3)	0.2963 (2)	0.5183 (2)	0.0226 (6)	
C9	−0.0066 (3)	0.5733 (2)	0.6191 (2)	0.0224 (6)	
C10	0.0675 (3)	0.1649 (2)	0.2990 (2)	0.0217 (6)	
C11	0.0668 (3)	−0.1207 (3)	0.2037 (3)	0.0276 (6)	
H11	0.1254	−0.1912	0.2025	0.033*	
C12	0.2288 (3)	0.3027 (2)	0.3068 (2)	0.0223 (6)	
C13	−0.0118 (3)	0.2112 (2)	0.3926 (2)	0.0213 (6)	
C14	0.1075 (3)	−0.0321 (3)	0.2480 (2)	0.0245 (6)	
H14	0.1952	−0.0418	0.2757	0.029*	
C15	−0.1602 (3)	0.1787 (2)	0.4738 (2)	0.0221 (6)	
C16	0.3274 (3)	0.1966 (2)	0.1389 (2)	0.0235 (6)	
C17	0.2780 (3)	0.1859 (3)	0.0443 (3)	0.0258 (6)	
H17	0.1721	0.1919	0.0499	0.031*	
C18	0.0215 (3)	0.0706 (2)	0.2524 (2)	0.0207 (6)	
C19	0.4833 (3)	0.1866 (3)	0.1293 (3)	0.0304 (7)	
H19	0.5189	0.1928	0.1930	0.036*	
C20	−0.3517 (3)	0.0349 (3)	0.5605 (3)	0.0310 (7)	
H20	−0.3834	−0.0413	0.5688	0.037*	
C21	−0.2565 (3)	0.2554 (3)	0.5369 (3)	0.0294 (7)	
H21	−0.2239	0.3308	0.5314	0.035*	
C22	0.3820 (3)	0.1667 (3)	−0.0578 (3)	0.0307 (7)	
H22	0.3474	0.1603	−0.1219	0.037*	
C23	−0.2089 (3)	0.0658 (3)	0.4899 (3)	0.0269 (6)	
H23	−0.1429	0.0097	0.4519	0.032*	
C24	0.3519 (3)	0.3845 (2)	0.2820 (2)	0.0234 (6)	
C25	0.4435 (4)	0.3824 (3)	0.3557 (3)	0.0347 (7)	
H25	0.4257	0.3288	0.4249	0.042*	
C26	−0.1052 (3)	0.0846 (3)	0.2092 (3)	0.0289 (7)	
H26	−0.1647	0.1546	0.2112	0.035*	
C27	−0.1446 (4)	−0.0030 (3)	0.1637 (3)	0.0365 (8)	
H27	−0.2305	0.0076	0.1339	0.044*	
C28	−0.4482 (3)	0.1143 (3)	0.6187 (3)	0.0334 (7)	
H28	−0.5469	0.0934	0.6656	0.040*	
C29	−0.0598 (4)	−0.1062 (3)	0.1611 (3)	0.0323 (7)	
H29	−0.0879	−0.1665	0.1305	0.039*	
C31	0.3810 (4)	0.4643 (3)	0.1820 (3)	0.0317 (7)	
H31	0.3192	0.4675	0.1314	0.038*	
C32	0.5360 (3)	0.1567 (3)	−0.0658 (3)	0.0327 (7)	
H32	0.6071	0.1424	−0.1353	0.039*	
C33	0.4987 (4)	0.5394 (3)	0.1546 (3)	0.0400 (8)	
H33	0.5175	0.5933	0.0856	0.048*	
C34	−0.3997 (3)	0.2246 (3)	0.6082 (3)	0.0338 (7)	
H34	−0.4640	0.2791	0.6496	0.041*	
C35	0.5869 (3)	0.1674 (3)	0.0263 (3)	0.0355 (8)	
H35	0.6930	0.1618	0.0198	0.043*	
C36	0.5888 (4)	0.5358 (3)	0.2279 (3)	0.0408 (8)	
H36	0.6702	0.5867	0.2090	0.049*	
C37	0.5606 (4)	0.4587 (3)	0.3276 (3)	0.0417 (8)	
H37	0.6218	0.4572	0.3782	0.050*	
Cl1	−0.0868 (8)	0.3795 (4)	−0.0111 (8)	0.117 (2)	0.50
Cl2	0.0383 (12)	0.5927 (6)	−0.0056 (8)	0.162 (4)	0.50
C38	0.0563 (14)	0.4593 (14)	−0.0020 (13)	0.094 (4)	0.50
H38A	0.0815	0.4261	0.0703	0.113*	0.50
H38B	0.1471	0.4448	−0.0651	0.113*	0.50

### Atomic displacement parameters (Å^2^)

**Table:**

	*U*^11^	*U*^22^	*U*^33^	*U*^12^	*U*^13^	*U*^23^
F1	0.0465 (10)	0.0238 (9)	0.0303 (9)	0.0017 (8)	−0.0210 (8)	−0.0035 (8)
F2	0.0434 (10)	0.0266 (9)	0.0272 (9)	0.0028 (7)	−0.0160 (8)	−0.0084 (8)
F3	0.0380 (10)	0.0196 (8)	0.0332 (10)	0.0042 (7)	−0.0123 (8)	−0.0041 (7)
C4	0.0216 (13)	0.0167 (12)	0.0233 (14)	−0.0045 (10)	−0.0064 (11)	−0.0008 (11)
C5	0.0219 (13)	0.0230 (14)	0.0217 (13)	−0.0038 (11)	−0.0060 (10)	−0.0005 (11)
C6	0.0190 (13)	0.0217 (13)	0.0243 (14)	−0.0023 (10)	−0.0060 (10)	−0.0040 (11)
C7	0.0228 (13)	0.0180 (13)	0.0248 (14)	−0.0029 (10)	−0.0041 (11)	−0.0047 (11)
C8	0.0229 (13)	0.0169 (13)	0.0269 (15)	−0.0019 (10)	−0.0063 (11)	−0.0010 (11)
C9	0.0235 (13)	0.0219 (14)	0.0221 (14)	−0.0036 (11)	−0.0064 (11)	−0.0023 (11)
C10	0.0183 (12)	0.0186 (13)	0.0283 (14)	−0.0002 (10)	−0.0072 (11)	−0.0032 (11)
C11	0.0273 (14)	0.0232 (15)	0.0300 (15)	−0.0016 (11)	−0.0019 (12)	−0.0081 (13)
C12	0.0214 (13)	0.0224 (14)	0.0226 (14)	−0.0018 (11)	−0.0060 (11)	−0.0027 (12)
C13	0.0205 (13)	0.0171 (13)	0.0259 (14)	−0.0025 (10)	−0.0063 (11)	−0.0021 (11)
C14	0.0207 (13)	0.0276 (15)	0.0253 (14)	0.0000 (11)	−0.0049 (11)	−0.0079 (12)
C15	0.0227 (13)	0.0213 (13)	0.0223 (13)	−0.0028 (10)	−0.0063 (11)	−0.0024 (11)
C16	0.0255 (14)	0.0176 (13)	0.0246 (14)	−0.0028 (11)	−0.0017 (11)	−0.0039 (11)
C17	0.0201 (13)	0.0286 (15)	0.0281 (15)	−0.0068 (11)	−0.0037 (11)	−0.0050 (12)
C18	0.0187 (12)	0.0223 (13)	0.0205 (13)	−0.0046 (10)	−0.0026 (10)	−0.0045 (11)
C19	0.0262 (15)	0.0327 (16)	0.0299 (16)	0.0008 (12)	−0.0075 (12)	0.0000 (13)
C20	0.0316 (15)	0.0295 (16)	0.0313 (16)	−0.0106 (12)	−0.0054 (13)	−0.0044 (13)
C21	0.0269 (15)	0.0244 (15)	0.0341 (16)	−0.0024 (11)	−0.0021 (12)	−0.0068 (13)
C22	0.0336 (16)	0.0306 (16)	0.0277 (16)	−0.0072 (13)	−0.0043 (13)	−0.0079 (13)
C23	0.0261 (14)	0.0240 (14)	0.0287 (15)	−0.0023 (11)	−0.0039 (12)	−0.0046 (12)
C24	0.0218 (13)	0.0210 (13)	0.0270 (14)	−0.0029 (10)	−0.0034 (11)	−0.0072 (12)
C25	0.0331 (16)	0.0360 (17)	0.0350 (17)	−0.0132 (13)	−0.0106 (13)	0.0023 (15)
C26	0.0291 (15)	0.0222 (14)	0.0386 (17)	−0.0040 (11)	−0.0155 (13)	−0.0016 (13)
C27	0.0411 (18)	0.0307 (17)	0.0441 (19)	−0.0085 (14)	−0.0243 (15)	0.0011 (15)
C28	0.0227 (14)	0.0416 (19)	0.0320 (16)	−0.0069 (13)	−0.0019 (12)	−0.0023 (14)
C29	0.0419 (18)	0.0266 (16)	0.0323 (16)	−0.0133 (13)	−0.0136 (14)	−0.0036 (14)
C31	0.0347 (16)	0.0268 (15)	0.0356 (17)	−0.0078 (13)	−0.0127 (13)	−0.0017 (14)
C32	0.0293 (15)	0.0350 (17)	0.0271 (15)	−0.0004 (13)	0.0043 (12)	−0.0077 (14)
C33	0.047 (2)	0.0312 (17)	0.0377 (19)	−0.0139 (15)	−0.0073 (15)	0.0023 (15)
C34	0.0295 (16)	0.0342 (17)	0.0331 (17)	0.0017 (13)	0.0001 (13)	−0.0095 (14)
C35	0.0223 (14)	0.0395 (19)	0.0370 (18)	0.0033 (13)	−0.0028 (13)	0.0027 (15)
C36	0.0344 (17)	0.0363 (18)	0.049 (2)	−0.0212 (14)	−0.0042 (15)	−0.0032 (16)
C37	0.0335 (17)	0.050 (2)	0.048 (2)	−0.0134 (15)	−0.0175 (15)	−0.0084 (18)
Cl1	0.127 (3)	0.065 (3)	0.176 (6)	0.009 (3)	−0.088 (3)	0.003 (3)
Cl2	0.279 (11)	0.087 (3)	0.127 (4)	−0.045 (5)	−0.064 (7)	−0.003 (3)
C38	0.072 (7)	0.122 (8)	0.096 (9)	0.045 (6)	−0.029 (7)	−0.055 (10)

### Geometric parameters (Å, °)

**Table:**

F1—C5	1.346 (3)	C20—C23	1.387 (4)
F2—C9	1.343 (3)	C20—H20	0.9500
F3—C8	1.344 (3)	C21—C34	1.392 (4)
C4—C9	1.335 (4)	C21—H21	0.9500
C4—C5	1.457 (4)	C22—C32	1.382 (4)
C4—C4^i^	1.478 (5)	C22—H22	0.9500
C5—C8	1.327 (4)	C23—H23	0.9500
C6—C12	1.343 (4)	C24—C31	1.388 (4)
C6—C16	1.487 (4)	C24—C25	1.400 (4)
C6—C10	1.492 (4)	C25—C37	1.389 (4)
C7—C8	1.494 (4)	C25—H25	0.9500
C7—C9^i^	1.511 (4)	C26—C27	1.381 (4)
C7—C12	1.541 (4)	C26—H26	0.9500
C7—C13	1.552 (4)	C27—C29	1.387 (5)
C9—C7^i^	1.511 (4)	C27—H27	0.9500
C10—C13	1.357 (4)	C28—C34	1.387 (5)
C10—C18	1.484 (4)	C28—H28	0.9500
C11—C14	1.388 (4)	C29—H29	0.9500
C11—C29	1.391 (4)	C31—C33	1.386 (4)
C11—H11	0.9500	C31—H31	0.9500
C12—C24	1.480 (4)	C32—C35	1.377 (5)
C13—C15	1.475 (4)	C32—H32	0.9500
C14—C18	1.388 (4)	C33—C36	1.382 (5)
C14—H14	0.9500	C33—H33	0.9500
C15—C21	1.388 (4)	C34—H34	0.9500
C15—C23	1.407 (4)	C35—H35	0.9500
C16—C19	1.396 (4)	C36—C37	1.368 (5)
C16—C17	1.397 (4)	C36—H36	0.9500
C17—C22	1.388 (4)	C37—H37	0.9500
C17—H17	0.9500	Cl1—C38	1.721 (16)
C18—C26	1.396 (4)	Cl2—C38	1.564 (17)
C19—C35	1.394 (4)	C38—H38A	0.9900
C19—H19	0.9500	C38—H38B	0.9900
C20—C28	1.382 (4)		
			
C9—C4—C5	124.5 (3)	C15—C21—C34	121.7 (3)
C9—C4—C4^i^	119.9 (3)	C15—C21—H21	119.1
C5—C4—C4^i^	115.5 (3)	C34—C21—H21	119.1
C8—C5—F1	119.4 (3)	C32—C22—C17	119.8 (3)
C8—C5—C4	122.8 (3)	C32—C22—H22	120.1
F1—C5—C4	117.8 (2)	C17—C22—H22	120.1
C12—C6—C16	125.7 (3)	C20—C23—C15	121.0 (3)
C12—C6—C10	110.2 (2)	C20—C23—H23	119.5
C16—C6—C10	124.1 (2)	C15—C23—H23	119.5
C8—C7—C9^i^	108.8 (2)	C31—C24—C25	118.4 (3)
C8—C7—C12	111.7 (2)	C31—C24—C12	119.8 (3)
C9^i^—C7—C12	107.0 (2)	C25—C24—C12	121.8 (3)
C8—C7—C13	114.2 (2)	C37—C25—C24	120.0 (3)
C9^i^—C7—C13	112.2 (2)	C37—C25—H25	120.0
C12—C7—C13	102.6 (2)	C24—C25—H25	120.0
C5—C8—F3	119.7 (3)	C27—C26—C18	120.4 (3)
C5—C8—C7	125.6 (3)	C27—C26—H26	119.8
F3—C8—C7	114.6 (2)	C18—C26—H26	119.8
C4—C9—F2	121.1 (2)	C26—C27—C29	120.5 (3)
C4—C9—C7^i^	127.2 (3)	C26—C27—H27	119.7
F2—C9—C7^i^	111.6 (2)	C29—C27—H27	119.7
C13—C10—C18	127.5 (2)	C20—C28—C34	119.4 (3)
C13—C10—C6	109.8 (2)	C20—C28—H28	120.3
C18—C10—C6	122.7 (2)	C34—C28—H28	120.3
C14—C11—C29	119.9 (3)	C27—C29—C11	119.5 (3)
C14—C11—H11	120.1	C27—C29—H29	120.2
C29—C11—H11	120.1	C11—C29—H29	120.2
C6—C12—C24	128.6 (3)	C33—C31—C24	121.0 (3)
C6—C12—C7	109.0 (2)	C33—C31—H31	119.5
C24—C12—C7	122.1 (2)	C24—C31—H31	119.5
C10—C13—C15	128.9 (2)	C35—C32—C22	120.3 (3)
C10—C13—C7	108.2 (2)	C35—C32—H32	119.8
C15—C13—C7	122.9 (2)	C22—C32—H32	119.8
C11—C14—C18	120.8 (3)	C36—C33—C31	119.9 (3)
C11—C14—H14	119.6	C36—C33—H33	120.0
C18—C14—H14	119.6	C31—C33—H33	120.0
C21—C15—C23	117.3 (3)	C28—C34—C21	119.9 (3)
C21—C15—C13	122.8 (3)	C28—C34—H34	120.0
C23—C15—C13	119.9 (3)	C21—C34—H34	120.0
C19—C16—C17	118.8 (3)	C32—C35—C19	120.3 (3)
C19—C16—C6	120.9 (3)	C32—C35—H35	119.9
C17—C16—C6	120.3 (2)	C19—C35—H35	119.9
C22—C17—C16	120.7 (3)	C37—C36—C33	119.8 (3)
C22—C17—H17	119.7	C37—C36—H36	120.1
C16—C17—H17	119.7	C33—C36—H36	120.1
C14—C18—C26	118.9 (3)	C36—C37—C25	120.9 (3)
C14—C18—C10	120.0 (2)	C36—C37—H37	119.6
C26—C18—C10	121.0 (2)	C25—C37—H37	119.6
C35—C19—C16	120.1 (3)	Cl2—C38—Cl1	122.2 (8)
C35—C19—H19	119.9	Cl2—C38—H38A	106.8
C16—C19—H19	119.9	Cl1—C38—H38A	106.8
C28—C20—C23	120.5 (3)	Cl2—C38—H38B	106.8
C28—C20—H20	119.7	Cl1—C38—H38B	106.8
C23—C20—H20	119.7	H38A—C38—H38B	106.6
			
C9—C4—C5—C8	179.9 (3)	C10—C13—C15—C23	26.1 (4)
C4^i^—C4—C5—C8	−2.1 (4)	C7—C13—C15—C23	−153.3 (3)
C9—C4—C5—F1	−1.2 (4)	C12—C6—C16—C19	45.4 (4)
C4^i^—C4—C5—F1	176.9 (3)	C10—C6—C16—C19	−137.8 (3)
F1—C5—C8—F3	−1.1 (4)	C12—C6—C16—C17	−132.5 (3)
C4—C5—C8—F3	177.8 (2)	C10—C6—C16—C17	44.3 (4)
F1—C5—C8—C7	−175.4 (2)	C19—C16—C17—C22	−0.6 (4)
C4—C5—C8—C7	3.5 (4)	C6—C16—C17—C22	177.4 (3)
C9^i^—C7—C8—C5	−4.0 (4)	C11—C14—C18—C26	−1.3 (4)
C12—C7—C8—C5	113.9 (3)	C11—C14—C18—C10	−179.4 (3)
C13—C7—C8—C5	−130.2 (3)	C13—C10—C18—C14	−117.4 (3)
C9^i^—C7—C8—F3	−178.5 (2)	C6—C10—C18—C14	61.8 (4)
C12—C7—C8—F3	−60.6 (3)	C13—C10—C18—C26	64.6 (4)
C13—C7—C8—F3	55.3 (3)	C6—C10—C18—C26	−116.2 (3)
C5—C4—C9—F2	−0.6 (4)	C17—C16—C19—C35	0.7 (4)
C4^i^—C4—C9—F2	−178.5 (3)	C6—C16—C19—C35	−177.3 (3)
C5—C4—C9—C7^i^	−178.9 (3)	C23—C15—C21—C34	−3.9 (4)
C4^i^—C4—C9—C7^i^	3.2 (5)	C13—C15—C21—C34	176.4 (3)
C12—C6—C10—C13	0.4 (3)	C16—C17—C22—C32	0.7 (5)
C16—C6—C10—C13	−176.8 (3)	C28—C20—C23—C15	−1.5 (5)
C12—C6—C10—C18	−178.9 (3)	C21—C15—C23—C20	4.1 (4)
C16—C6—C10—C18	3.9 (4)	C13—C15—C23—C20	−176.1 (3)
C16—C6—C12—C24	1.1 (5)	C6—C12—C24—C31	61.1 (4)
C10—C6—C12—C24	−176.1 (3)	C7—C12—C24—C31	−111.4 (3)
C16—C6—C12—C7	174.3 (3)	C6—C12—C24—C25	−117.3 (4)
C10—C6—C12—C7	−2.8 (3)	C7—C12—C24—C25	70.3 (4)
C8—C7—C12—C6	126.6 (3)	C31—C24—C25—C37	−0.3 (5)
C9^i^—C7—C12—C6	−114.4 (3)	C12—C24—C25—C37	178.1 (3)
C13—C7—C12—C6	3.9 (3)	C14—C18—C26—C27	0.5 (4)
C8—C7—C12—C24	−59.6 (3)	C10—C18—C26—C27	178.5 (3)
C9^i^—C7—C12—C24	59.3 (3)	C18—C26—C27—C29	0.6 (5)
C13—C7—C12—C24	177.6 (2)	C23—C20—C28—C34	−1.5 (5)
C18—C10—C13—C15	2.0 (5)	C26—C27—C29—C11	−0.7 (5)
C6—C10—C13—C15	−177.3 (3)	C14—C11—C29—C27	−0.2 (5)
C18—C10—C13—C7	−178.5 (3)	C25—C24—C31—C33	0.7 (5)
C6—C10—C13—C7	2.1 (3)	C12—C24—C31—C33	−177.8 (3)
C8—C7—C13—C10	−124.6 (3)	C17—C22—C32—C35	−0.9 (5)
C9^i^—C7—C13—C10	111.0 (3)	C24—C31—C33—C36	−0.3 (5)
C12—C7—C13—C10	−3.6 (3)	C20—C28—C34—C21	1.8 (5)
C8—C7—C13—C15	54.9 (3)	C15—C21—C34—C28	1.0 (5)
C9^i^—C7—C13—C15	−69.5 (3)	C22—C32—C35—C19	1.1 (5)
C12—C7—C13—C15	175.9 (2)	C16—C19—C35—C32	−0.9 (5)
C29—C11—C14—C18	1.2 (4)	C31—C33—C36—C37	−0.5 (6)
C10—C13—C15—C21	−154.1 (3)	C33—C36—C37—C25	0.9 (6)
C7—C13—C15—C21	26.5 (4)	C24—C25—C37—C36	−0.5 (5)

Symmetry codes: (i) −*x*, −*y*+1, −*z*+1.
